# Ice-recrystallization inhibiting polymers protect proteins against freeze-stress and enable glycerol-free cryostorage†
†Electronic supplementary information (ESI) available. See DOI: 10.1039/c8mh00727f


**DOI:** 10.1039/c8mh00727f

**Published:** 2018-11-08

**Authors:** Daniel E. Mitchell, Alice E. R. Fayter, Robert C. Deller, Muhammad Hasan, Jose Gutierrez-Marcos, Matthew I. Gibson

**Affiliations:** a Department of Chemistry , University of Warwick , Coventry , CV47AL , UK . Email: M.i.gibson@warwick.ac.uk; b School of Life Sciences , University of Warwick , Coventry , CV47AL , UK; c Warwick Medical School , University of Warwick , CV47AL , UK

## Abstract

Proteins are ubiquitous in molecular biotechnology, biotechnology and as therapeutics, but there are significant challenges in their storage and distribution, with freezing often required. This is traditionally achieved by the addition of cryoprotective agents such as glycerol (or trehalose) or covalent modification of mutated proteins with cryoprotectants. Here, ice recrystallization inhibiting polymers, inspired by antifreeze proteins, are used synergistically with poly(ethylene glycol) as an alternative to glycerol. The primary mechanism of action appears to be preventing irreversible aggregation due to ice growth. The polymer formulation is successfully used to cryopreserve a range of important proteins including insulin, Taq DNA polymerase and an IgG antibody. The polymers do not require covalent conjugation, nor modification of the protein and are already used in a wide range of biomedical applications, which will facilitate translation to a range of biologics.

## Abstract 2

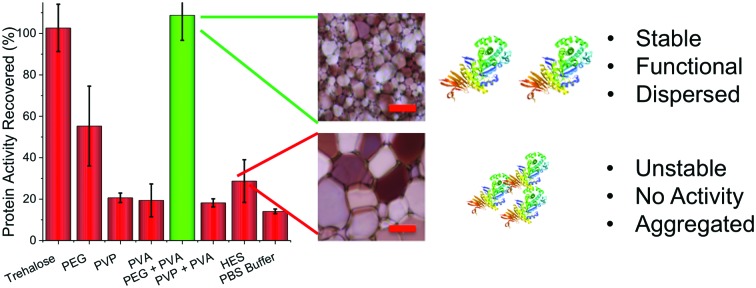
Antifreeze-protein mimic polymers are shown to enable solvent-free storage of important proteins for therapy and biotechnology by modulating ice growth.

##

Conceptual insights

The current method used in biochemical, molecular biology, protein engineering and therapeutic labs for freezing proteins is often based on adding organic solvents to modulate ice formation and stabilise the proteins. Our work takes a disruptive approach, using antifreeze-protein mimetic polymers, which are extremely potent inhibitors of ice growth, to enable protein cryostorage and eliminate the need for organic solvents whilst recovering active protein. In recent years covalent polymer/protein conjugates have been successfully used to freeze proteins, but this can be complex, reduce protein function and crucially generates a new molecular entity which must be tested. In our unique approach, we identified that irreversible protein aggregation due to ice crystal growth is a major cause of cryo-damage and that if we prevent this, the proteins retain activity. We have formulated polymer additives which modulate ice growth and enable quantitative recovery of a range of important proteins without needing conjugation. This work shows that biomimetic materials chemistry can be applied to a real clinical problem to generate unique solutions which will enable new therapies.

## Introduction

Proteins are ubiquitous as laboratory reagents, biocatalysts, medicines and as food supplements. For example, in the 1980's insulin became the first protein therapeutic, and now >100 are approved in the European Union and the USA.^1^,^2^ Antibody therapies in particular have grown and are now used for treatment of cancer^3^ and inflammatory diseases,^4^ and are the fastest growing class of therapeutics.^5^ A major challenge, however, is the limited storage lifetime of proteins, with degradation being a major issue.^6^,^7^ Environmental stresses such as temperature, sunlight and dehydration are all crucial deactivation factors that may affect the chemical and physical stability of proteins which along with irreversible aggregation result in inactivated proteins.^2^,^8^ Current solutions to this challenge include lyophilization or direct freezing in solution with the addition of large concentrations of osmolytes that make unfolding thermodynamically less favourable,^9^ though more recently spray drying and vacuum foam drying have also been introduced.^10^,^11^ Whilst these methods are successful, there are post-thaw issues associated with the compatibility of high concentrations of osmolytes used (*e.g.* 10–20% glycerol). This can include sample viscosity or toxicity,^12^ interference with colorimetric assays, affecting protein–protein interactions or subsequent issues relating to cytotoxicity for studies into protein–cell interactions.^13^–^15^ Hence, direct therapeutic injection or use in biochemical assays may often necessitate an additional purification/dilution step. Tibbitt *et al.* have shown that the reversible encapsulation of proteins into photo-reversible hydrogel networks protects against thermal stress by preventing aggregation,^16^ and encapsulation in zwitterionic gels has provided *in vivo* stability.^17^ Trehalose has emerged as an excellent stabilizer/osmolyte and is widely used as a cryoprotectant.^9^,^18^ Maynard and co-workers have developed trehalose polymers, which when covalently conjugated to enzymes can protect them from heat and cold shock. Intriguingly, only a single polymer per protein is required, thus demonstrating the potential use of new polymeric compounds as excipients.^19^,^20^ However, the conjugation process can reduce activity relative to free protein and depending on the protein site-specific mutations may be necessary.^21^,^22^ There is also some evidence that dietary trehalose can increase the virulence of *C. difficile* infections.^23^

Antifreeze (glyco)proteins (AF(G)P)s from polar fish provide protection against ice formation and growth in hypothermal conditions and have the most potent ice recrystallization inhibition (IRI) activity known.^24^ Synthetic IRI-active compounds developed by Ben *et al.*^25^ have been shown to enhance cellular recovery during cryopreservation.^26^ Gibson and co-workers have developed synthetic polymers as mimetics of AF(G)Ps,^27^,^28^ which have potent IRI activity and can be employed in solvent-free cryopreservation.^29^–^32^ The most potent IRI-active polymer to date is PVA (poly(vinyl alcohol)). PVA is a particularly appealing additive as it is widely used in pharmaceutical formulations and is an FDA approved food additive.^33^ Mitchell *et al.* found that the reversible aggregation of gold nanoparticles during freezing could be modulated by addition of PVA.^34^ By slowing the rate of ice growth, the effective surface area of the ice crystals is increased (*i.e.* more small crystals) and hence the nanoparticles could not approach each other to aggregate. We therefore hypothesized that IRI-active compounds might prevent protein aggregation during ice-growth induced stress, which normally leads to denaturation/deactivation. This effect could in turn be exploited in the solvent-free storage proteins.

## Results & discussion

**Fig. 1:**
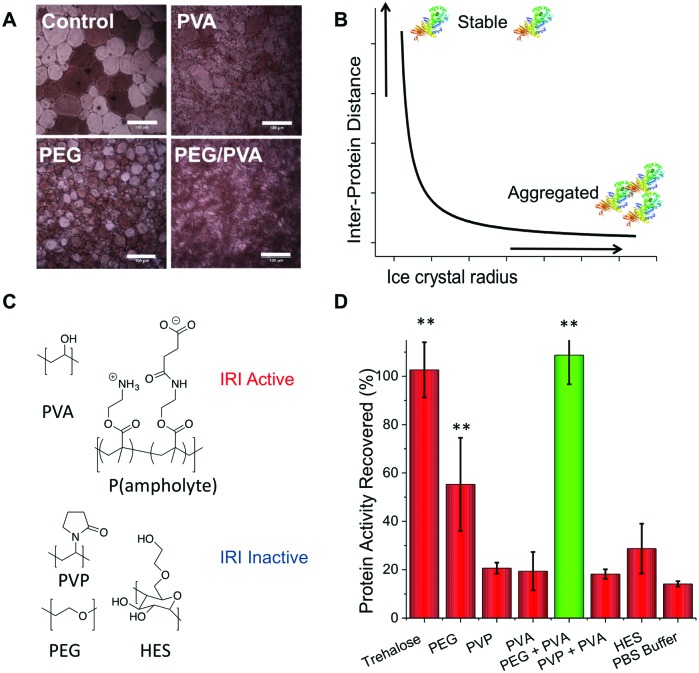
Ice recrystallization inhibiting polymer mediated protein storage. (A) Example ice crystal wafers grown with and without added PVA. Scale bar – 100 μm; (B) Schematic showing protein aggregation (concentration) changes as a function of ice crystal size; (C) polymers used in this study; (D) recovery of β-Gal activity after freezing for 3 days at –20 °C, as % of fresh, unfrozen protein. Error bars are S. D. from a minimum of 6 repeats, ** represents *p* < 0.01 relative to PBS buffer control. PEG, PVP, trehalose and HES at concentration of 100 mg mL^–1^, PVA at 1 mg mL^–1^.

The aim of this study was to investigate the utility of IRI-active polymers as ‘polymer-only’ protein stabilizing agents for medically and biotechnologically important proteins. IRI activity was studied using a modified splat assay. This showed that mean length of ice crystals grown and held at sub-zero temperatures (–8 °C) in the presence of PVA are significantly smaller (*x[combining macron]* = 15 μm) than those grown without it (*x[combining macron]* = 124 μm) (Fig. 1A). Smaller crystals indicate more IRI-activity. In a normal frozen formulation any proteins present would be phase separated to the surface of ice crystals, producing a freeze concentrated liquid and an ice phase. As the surface area of ice decreases, the proteins at the surface should become more concentrated, and hence the likelihood of aggregation will also increase. (Fig. 1B shows schematic of this process).^35^ Here, we investigate the ability of IRI-active polymers as non-covalent protein stabilizers to prevent these deleterious effects. An initial screen for cryoprotection/damage was conducted using bacterial β-galactosidase (β-Gal).

β-Gal was frozen at –20 °C for 3 days in the presence of trehalose (as a positive control), PVA or various polymers (HES, PVP, PEG) which are known to have no IRI activity.^28^,^29^ Activity was tested after thawing at 20 °C (Fig. 1D).

As expected, trehalose protected β-Gal activity during freezing.^9^ All other additives failed to protect when used individually, apart from PEG which gave some protection (Fig. 1D). It should be noted that PEG has known cryoprotectant properties at sufficiently high concentrations, making it the ideal choice for a synergistic cryoprotectant.^36^ When PEG (100 mg mL^–1^) and PVA (1 mg mL^–1^) were combined, a synergistic cryoprotective effect was observed, reaching values equivalent to trehalose (Fig. 1D). The combination of PEG/PVA appears to be unique since PVP/PVA mixtures were no different from individual additives. Variable concentration studies (ESI†) show that the PEG concentration could be lowered (at constant [PVA]) to as low as 50 mg mL^–1^ (∼5 wt%) without affecting recovery, but below 30 mg mL^–1^ there was no protection. We have previously reported that a secondary water soluble polymer is required when using IRI-active polymers for cellular cryopreservation, agreeing with the observations here.^29^ PEG has widely been found to have a stabilization effect on various proteins, and is used to improve delivery in pharmaceutical applications.^37^ Several studies suggest that PEG chains can interact with the protein surface reducing solvent accessible area,^38^ and prevent unfolding and aggregation through molecular crowding.^39^ These stabilization effects would also be useful in cryopreservation, preventing aggregation caused by reduced liquid water volume and any denaturing caused by low temperatures.

**Fig. 2:**
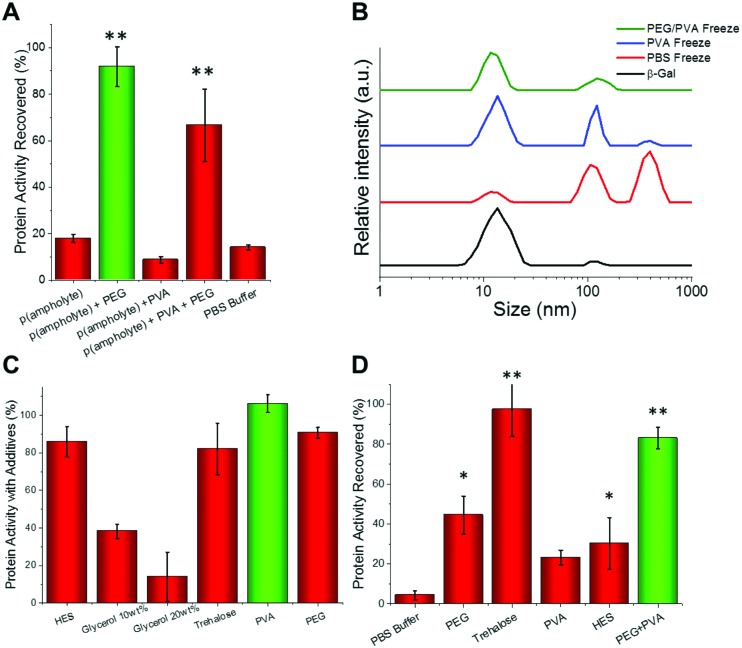
(A) Protein freeze/thaw recovery upon addition of p(ampholyte) following β-Gal storage for 3 days at –20 °C, as % of fresh, unfrozen protein. Concentrations as in Fig. 1, but p(ampholyte) used at 30 mg mL^–1^. (B) DLS analysis of β-Gal aggregation post freezing compared to fresh protein (black line). (C) β-Gal activity after 1 hour incubation in the cryostorage solutions without freeze/thaw; (D) β-Gal recovery after 4 weeks storage at –80 °C. Error bars are S. D. from minimum of 3 repeats.

We hypothesised that the role of PVA in enhancing protein cryostorage may be due to its IRI-activity. To test this hypothesis, we tested another IRI-active polymer (p(ampholyte)) developed in our laboratory. Because p(ampholyte) is less IRI active, we employed a higher concentration to achieve equal IRI activity comparable to that of PVA at 1 mg mL^–1^. Under identical conditions, we found that p(ampholyte) and PVA displayed identical cryoprotectant properties (Fig. 2A). This data corroborates the hypothesis that ice growth is a cause of protein deactivation and that the rational design of IRI-macromolecules may be an effective strategy for the discovery of new protein stabilizers and be complementary to small molecular cryoprotectants which function by different mechanisms. To investigate if preventing protein aggregation was a critical factor for protein cryoprotection, we employed dynamic light scattering (DLS). When β-Gal was freeze/thawed in phosphate-buffered saline (PBS) alone, large aggregates could be seen (>500 nm in diameter) (Fig. 2B). Similarly, large aggregates were also observed when only PVA polymers were employed. Conversely, PEG/PVA mixtures prevented all freeze-induced aggregation (Fig. 2B).

Our bio-inspired macromolecules have some advantages over small-molecule cryoprotectants as they have low cytotoxicity, high biocompatibility (FDA approved/GRAS status for several applications),^33^,^40^ and at equal mass concentrations have lower molar concentration thus imposing less osmotic stress. To confirm that our polymers are passive additives, we incubated β-Gal with different concentrations of cryoprotectants and tested activity (Fig. 2C). Glycerol solutions significantly impaired protein function or the assay output, but PEG, PVA and trehalose had little impact on protein function, thus indicating that they are biologically inert and may not need removing before use, providing an alternative to solvent based approaches, where dialysis is typically required post-thaw.

Since under standard laboratory conditions proteins are normally stored at –80 °C through long periods, we decided to test the levels of protein activity at this temperature. The PEG/PVA formulation enabled recovery of β-Gal activity comparable to that of trehalose (Fig. 2D).

**Fig. 3:**
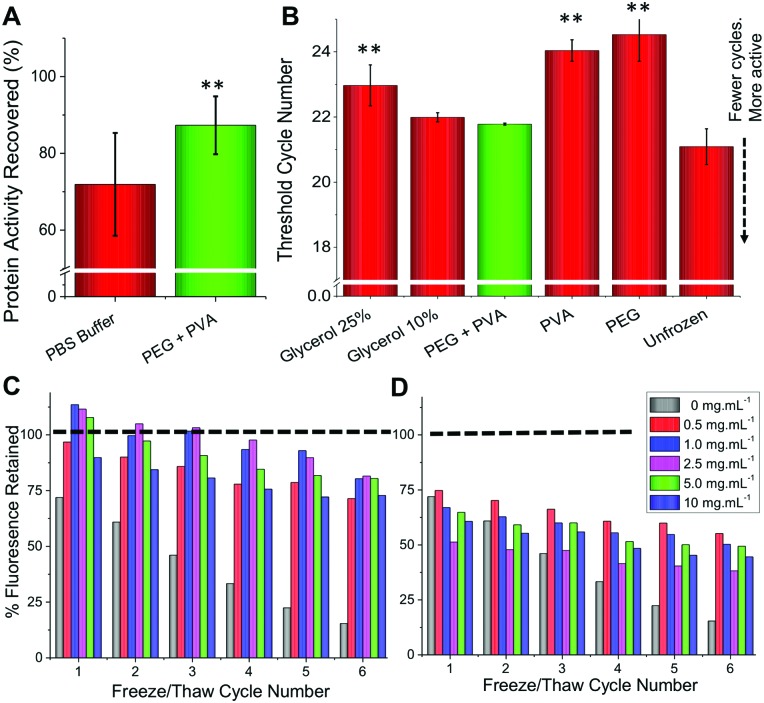
(A) Glucose oxidase recovery after 3 days storage at –20 °C; (B) Taq polymerase activity recovery, after 3 days storage at –20 °C, expressed as number of threshold cycles (lower is more active). ** represents *p* < 0.01 compared to control, error bars are from a minimum of 6 repeats. C + D show fluorescence recovery of GFP after freeze (–20 °C)/thaw (27 °C). All solutions containing 100 mg mL^–1^ PEG plus PVA concentration indicated in the insert. (C) PVA 10 kDa; (D) PVA 23 kDa.

To ensure these observations were not unique to a single enzyme we set out to study a range of other proteins using this methodology. Glucose oxidase (GOx) is widely used in sensing, food industry and in molecular biology^41^ whilst hyperthermophylic DNA polymerase from *Thermus aquaticus* (Taq) is commonly used in diagnostics and research for the amplification of DNA through the polymerase chain reaction (PCR) that underpins modern genomic analyses.^42^ We found that GOx was relatively stable after freeze/thawing in only PBS, however, addition of IRI-active polymers did increase the recovered enzymatic activity (Fig. 3A). To assess the activity of Taq after freeze/thawing, we used quantitative PCR (qPCR) and determined the number of cycles to reach a threshold level; fewer cycles means more activity. We observed that PEG/PVA improved stability to the extent that it was comparable to fresh recombinantly-expressed enzyme (Fig. 3B).

Encouraged by the above results we proceeded to more closely reproduce laboratory or clinical settings where protein samples are often removed and replaced from/into a freezer, a freeze/thaw cycle assay was developed as a robust test of the technology. To enable continual monitoring of the same sample through many freeze/thaw cycles, recombinant green fluorescent protein (GFP) was used. Upon denaturation, the fluorescence decreases providing a convenient readout. Fig. 3C shows the recovery of fluorescence following 6 freeze (–20 °C) and thaw (27 °C) cycles. For PEG alone, there was a dramatic reduction in fluorescence with only 15% function retained after 6 cycles. Addition of 1 mg mL^–1^ PVA to PEG was found to be optimal, enabling >75% activity retention after 6 freeze/thaw cycles. Higher concentrations of PVA were found to be detrimental as was the use of higher molecular weight PVA (23 kDa) (Fig. 3D). We hypothesize that this is due to dynamic ice shaping – a common side effect of antifreeze proteins, which is known to compromise cell cryopreservation.^32^,^43^ This highlights the delicate balance of IRI-activity with ice shaping and that the actual polymer composition is crucial to success.

**Fig. 4:**
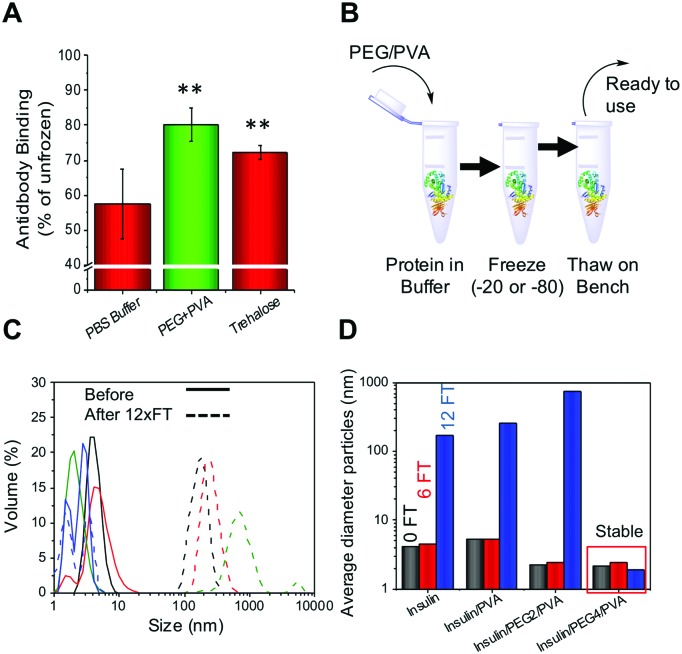
(A) Rabbit IgG activity recovered after 3 days storage at –20 °C relative to fresh antibody in a surface binding assay. ** represents *p* < 0.01, error bars are from a minimum of 6 repeats; (B) schematic showing ease of freezing methodology; (C) DLS curves of insulin before (solid) and after (dashed) 12 freeze/thaw cycles. Black = insulin only, Red = insulin + PVA, Green = +2 kDa PEG + PVA, Blue = + 4 kDa PEG + PVA. IN all cases PEG = 100 mg mL^–1^ and PVA =1 mg mL^–1^. (D) Average size of insulin aggregates after 6 or 12 FT cycles in indicated conditions.

To determine if this methodology could be used for therapeutic proteins as well as those described above, an antibody (rabbit IgG) and insulin were both tested. Antibodies are widely used in diagnostics (*e.g.* ELISA) and molecular biology as well as in emerging therapeutics, but maintaining their function upon storage is challenging. Rabbit IgG purified extract was stored at –20 °C for 3 days and function determined using an ELISA-based assay. As with all the other proteins tested the PEG/PVA mixture greatly enhanced IgG activity (>80%) and the recovery level was superior to that of trehalose (Fig. 4A). Insulin, a biologic therapeutic essential for managing diabetes, is deactivated upon liquid storage by simple agitation or by irreversible aggregation. Dynamic light scattering was therefore employed to probe the prevention of irreversible insulin aggregation upon freeze/thaw using a range of conditions (Fig. 4C). Again the PEG/PVA formulation prevented aggregation more so than PVA alone. Different molecular weight PEGs were considered, 4 kDa and 2 kDa, showing that 4 kDa PEG specifically, in combination with PVA protects insulin from aggregation and thus inactivity. This implies certain molecular weights are important for a solvent-free, polymer only cryopreservation formulation.

## Conclusions

In conclusion, we show that bio-inspired ice-recrystallization inhibiting polymers present a materials-chemistry solution to therapeutic protein storage, as an alternative to the traditional solvent-based approach. The formulation is shown to match the performance of trehalose, and offers some advantages over glycerol storage. The mechanism of action appears to be prevention of irreversible aggregation, with a secondary hydrophilic polymer (PEG) being essential, which is distinct from how small molecule cryoprotectants function. This is advantageous over current solvent based techniques, but also as an alternative to emerging covalent polymer–protein conjugation approaches. Our additives, PEG and PVA, are widely used in pharmaceutical products, are available as clinical-grade materials, are biocompatible and non-immunogenic, and are compatible with current biologics, with no additional bio-conjugation being required. These antifreeze protein-inspired polymers may be useful tools for protein storage in both biotechnology research and healthcare.

## Experimental

### Protein freezing

Samples were made in triplicate at the appropriate concentrations and frozen in 1.5 mL eppendorf tubes by directly placing in a freezer either at –20 °C or –80 °C. The samples were then held at this temperature for the indicated time period and then thawed at 20 °C.

### β-Gal activity

Determined by a colorimetric assay involving the use of *o*-nitrophenyl-β-d-galactoside (ONPG). Briefly aliquots of 30 μL of 4 mg mL^–1^ ONPG were added to wells of a 96 well plate containing 50 μL of 20 μg mL^–1^*β*-Gal solution. This was then incubated at room temperature for 5 minutes and quenched by addition of 50 μL of 1 M Na_2_CO_3_ solution. Absorbance was measured at 420 nm. All other methods are in the ESI.†

## Data access statement

The research data supporting this publication can be found at http://wrap.warwick.ac.uk.

## Conflicts of interest

MIG, DEM, RCD, AERF, MH are named inventors on patent filings relating to this research.

## Supplementary Material

Supplementary information
