# Supplementary material for: Ice-recrystallization inhibiting polymers protect proteins against freeze-stress and enable glycerol-free cryostorage
Source: Mater Horiz. 2018 Nov 8;6(2):364–8. doi: 10.1039/c8mh00727f (PMC6394881; doi:10.1039/c8mh00727f)
Supplement: Supplementary file 1 [file MH-006-C8MH00727F-s001.pdf]

Electronic Supplementary Material (ESI) for Materials Horizons.
This journal is © The Royal Society of Chemistry 2018

Supplementary Information for

Ice-Recrystallization Inhibiting Polymers Protect Proteins Against Freeze-Stress and Enable Glycerol-Free Cryostorage

Daniel E. Mitchell,[a,d] Alice E.R. Fayter,[a] Robert C. Deller,[a] Muhammad Hasan,[a] Jose Gutierrez-Marcos[c] and Matthew I. Gibson[a,b *]

[a] Department of Chemistry, University of Warwick, Gibbet Hill Road, Coventry, CV4 7AL, UK

[b] Warwick Medical School, University of Warwick, Gibbet Hill Road, Coventry, CV4 7AL, UK.

[c] School of Life sciences, University of Warwick, Gibbett Hill Road, Coventry, CV4 7AL, UK.

[d] Department of Chemistry, University of Sheffield, Sheffield, S3 7HF

Corresponding Author Email: m.i.gibson@warwick.ac.uk

Experimental Section

Materials

β-galactosidase (β-Gal), glucose-oxidase (GOx), o-dianosidine, glucose, horse radish peroxidase (HRP), o-nitrophenyl-β-D-galactoside (ONPG), ethanol, poly(vinyl pyrolidone) (PVP, 5 kDa), succinic anhydride, aminethyl methacrylate, insulin, rabbit IgG, poly(ethylene glycol) (PEG) (2.5, kDa 4kDa) and PVA (10, 23 and 30 kDa) and trehalose were purchased from Sigma-Aldrich. EvaGreen dye was purchased from VWR chemicals and M13 primers, dNTPs, PCR buffer (including $MgCl_2$) were purchased from Invitrogen. Rabbit IgG assay kit and Taq polymerase were purchased from Life technologies. cDNA was provided by the lab of Jose Gutierrez-Marcos (University of Warwick Life Sciences department). Phosphate-buffered saline (PBS) solution was prepared using preformulated tablets (Sigma-Aldrich) in 200 mL of Milli-Q water (>18.2 Ω mean resistivity) to give [NaCl] = 0.138 M, [KCl] = 0.0027 M, and pH 7.4. Taq polymerase was dialysed against glycerol free Taq buffer prior to use using Amicon 0.5 mL centrifugal filters (Merck, Irl) [Tris-HCl] = 10 mM, [KCl] = 100 mM, [DTT] = 1mM, [EDTA] = 0.1 mM, 0.5 wt % Tween 20, 0.5 wt % Triton X. Poly(aminoethyl methacrylate) (PAEMA, 32 kDa) functionalised with approximately 50 % COOH groups (PAEMA-co-SA) were synthesized as detailed previously. [1] All reagents were used as received unless otherwise stated.

Physical and analytical methods

Absorbance spectroscopy was undertaken using a SynergyHT multi-mode microplate reader (BioTek UK, Bedfordshire, UK). Quantitative polymerase chain reaction (QPCR) was carried out on a real-time PCR detection system while a thermocycler was used for standard PCR reactions. PCR was undertaken using the following protocol,

initiation at 94 °C for 1 minute, denaturation at 94 °C for 20 seconds, annealing at 56 °C for 20 seconds and elongation at 72 °C for 30 seconds. Twenty-five cycles were used and followed by a final elongation at 72 °C for 5 minutes. Circular Dichroism (CD) spectra were recorded on a spectropolarimeter (Jasco J-720, Jasco UK) using a data interval of 0.2 nm. The spectrum was measured 16 times and averaged. The spectrum of a blank sample containing only buffer or the appropriate cryoprotectant was then subtracted giving a final spectrum for each protein. Dynamic light scattering was undertaken on a Malvern Zetasizer Nano ZS.

β-galactosidase assay

β-Gal activity was determined by a colorimetric assay involving the use of ONPG. Aliquots of 30 μL of 4 mg.mL^{-1} ONPG were added to wells of a 96 well plate containing 50 μL of 20 μg.mL^{-1} β-Gal solution. This was then incubated at room temperature for 5 minutes and quenched by addition of 50 μL of 1M Na_2CO_3 solution. Absorbance was measured at 420 nm.

Glucose Oxidase assay

Activity of GOx was determined using the oxidation of o-dianosidine through a peroxidase-coupled reaction.[2] Briefly 2.5 mg of o-dianosidine was dissolved in 2 mL of ethanol, which was further diluted by the addition of 8 mL of PBS buffer resulting in a 10 mL stock solution. A Horseradish peroxidase (HRP) stock solution of 100 μg.mL^{-1} was prepared by dissolving HRP into a solution of 18 % w/w glucose in distilled water, while GOx samples were prepared at a concentration of 2 μg.mL^{-1}. 30 μL of GOx sample was added to 30 μL of HRP solution into separate wells of a 96 well

plate, then 150 μL of o-dianosidine was added and the mixture was incubated for 3 minutes. Finally, absorbance was measured at 450 nm.

Quantitative polymerase chain reaction assay

Cryoprotectants at required concentrations were added to Taq in the appropriate buffer solution in 20 μL volumes. QPCR was undertaken using standard protocols. EvaGreen dye was used as the DNA-binding fluorescent dye, sample volumes were 20 μL. Briefly, samples of 2.5 μL PCR buffer, 1 μL dNTPs, 1.5 μL Eva Green fluorescent dye, 1 μL forward and reverse primers, 1 μL of Taq at 1.25 U. μL^{-1} and 12 μL PCR water, were prepared. Samples were tested in triplicate with three dilutions of template DNA at 20, 10 and 5 ng, with appropriate positive and negative controls. Primers and template DNA were provided by the Gutierrez-Marcos lab (Department of Life Sciences, University of Warwick) from a previous PCR experiment that was found to be successful. The threshold cycle for successful amplification was achieved when the fluorescence becomes statistically significant above a baseline value. The baseline was set measuring average fluorescence between cycles 1-15 (1 cycle = denaturation, annealing and elongation).

Recombinant Expression and Purification of GFP

A pWALDO plasmid encoding for a hexahistidine-tagged GFP was kindly provided by Elizabeth Fullam (Warwick University, Coventry, UK). The plasmid was transformed into competent *Escherichia coli* BL21(DE3) cells (New England Biolabs). A colony was selected to inoculate 50 mL of LB-medium containing 100 $\mu g.mL^{-1}$ ampicillin and was grown overnight at 37 °C under continuous shaking of 180 rpm. The following day, 5 mL of the preculture was added to 500 mL of LB-medium in a 2 L Erlenmeyer

flask and grown at 37 °C for 4 hours with a shaking speed of 180 rpm. The temperature was then reduced to 16 °C and the cells incubated for another hour before adding IPTG to a final concentration of 1 mM. The overexpression of the protein was allowed to take place overnight following which the cells were centrifuged at 4000 *g* for 30 minutes at 4 °C.

Pelleted cells were resuspended in PBS supplemented with Pierce protease inhibitor mini-tablets. The suspension was passed through a STANSTED 'Pressure Cell' FPG12800 homogenizer in order to lyse the cells. The cell lysate was centrifuged at 14,000 *g* and the supernatant applied to an IMAC Sepharose 6 Fast Flow (GE Healthcare) column charged with Ni(II) ions and pre-equilibrated with PBS. The column was washed with 10 column volumes of 20 mM imidazole in PBS followed by 5 column volumes of 50 mM imidazole in PBS. Bound GFP was eluted using 250 mM (or 1000 mM) Imidazole in PBS. Imidazole was removed from the fractions containing GFP using PD10 desalting columns (GE Healthcare). Purity was estimated using SDS-PAGE and protein concentration determined using Thermo Scientific Pierce BCA assay kit. Various volumes of the GFP containing PBS solution were aliquoted into 1.5 mL microcentrifuge tubes and snap-frozen in liquid nitrogen to store at -80 °C till required.

Green Fluorescence Protein Stability Assay

200 μL of 6.89 $mg.mL^{-1}$ green fluorescent protein (GFP) was diluted in 39.8 mL PBS buffer resulting in a stock solution of 0.034 $mg.mL^{-1}$. PEG and PVA were dissolved in 2 mL of the stock solution to make different samples with final concentrations of 100 $mg.mL^{-1}$ PEG and 0.5, 1, 2.5, 5 and 10 $mg.mL^{-1}$ PVA. This was repeated for all 3 different molecular weights of PVA tested. Aliquots of 80 μL of the GFP/PEG/PVA

solution were pipetted into wells of a black 96 well plate and fluorescence recorded at 27 °C. Fluorescence intensity was compared to that of a GFP/PEG solution with a 100 $mg.mL^{-1}$ concentration. The plates were placed in a freezer at -20 °C until frozen and then thawed in an Eppendorf SmartBlock^{TM} at 27 °C for 10 minutes. The above freeze-thaw cycle was repeated 6 times with the fluorescence of the samples recorded after each thaw. Fluorescence excitation was measured at 485/20 nm and emission at 528/20 nm.

Insulin freeze-thaw assay

1 mL of 10.5 $mg.mL^{-1}$ insulin was diluted in 19 mL PBS buffer resulting in a stock solution of 0.525 $mg.mL^{-1}$. PEG and PVA were dissolved in the stock solution to make different samples with final concentrations of 100 $mg.mL^{-1}$ 4 kDa PEG, 50 $mg.mL^{-1}$ 2 kDa PEG and 1 $mg.mL^{-1}$ PVA. Insulin in PBS buffer was used as a control against solutions of Insulin/PVA, Insulin/PEG2/PVA and Insulin/PEG4/PVA. The samples were placed in a freezer at -20 °C for 1 hour until frozen and then thawed in an Eppendorf SmartBlock^{TM} at 37 °C for 15 minutes. The above freeze-thaw cycle was repeated for 6 and then 12 thaws before hydrodynamic diameter being measured by dynamic light scattering.

Rabbit IgG Assay

Activity of rabbit IgG was determined using an "Easy-Titer rabbit IgG assay kit (Life Technologies). 125 μL Solutions of 125 $\mu g.mL^{-1}$ of IgG were prepared and frozen for 4 days. Upon thawing, 20 μL of IgG sensitized beads were pipetted into wells of a 96 well plate and 20 μL of IgG solution was added. The plate was then incubated under

shaking for 5 minutes at room temperature after which 100 μL of blocking buffer was added and the plate was incubated for a further 5 minutes. Absorbance was measured at 405 nm using a plate reader, and samples were compared to a freshly made up positive control.

Freeze-thaw methodology

Samples were made in triplicate at the appropriate concentrations and frozen by placing in a freezer either at -20 °C or -80 °C. The samples were then held at this temperature within the freezer for the appropriate amount of time and then thawed on the bench top.

Additional Data

Serial Dilutions of Cryoprotectants

To determine the optimal concentrations of the excipient mixture, a range of concentrations of PEG/PVA mixtures were tested for β-Gal storage, Figure S1.

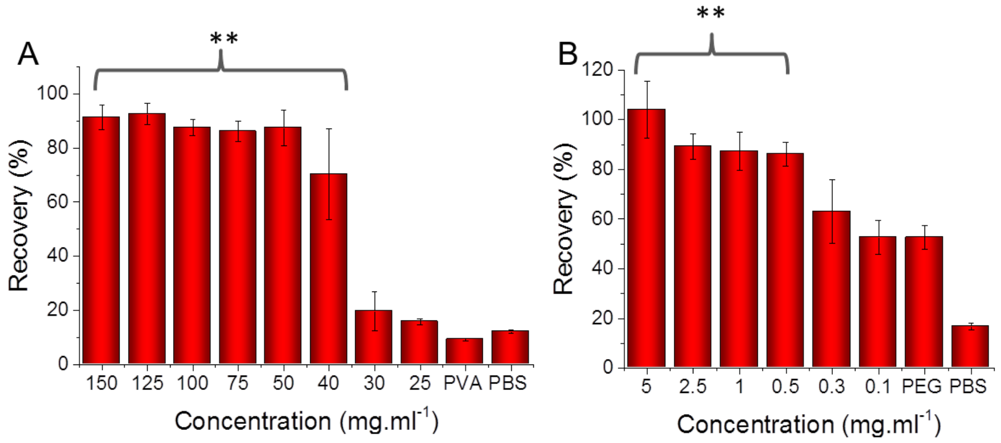

Figure S1. Dilutions of PEG and PVA frozen and stored for 3 days at -20 °C. A.) Serial dilution of PEG, with 1 mg.mL^{-1} PVA; B) Serial dilution of PVA, with 100 mg.mL^{-1} PEG. ** Represents $p < 0.01$ relative to PBS buffer, error bars are calculated from minimum of 6 repeats. Recovery expressed as a percentage of fresh unfrozen β-Gal.

Figure S1 shows that recovery drops significantly when PEG concentration is reduced below 50 mg.mL^{-1} (in the presence of 1 mg.mL^{-1} PVA) and recovery has plateaued at 100 mg.mL^{-1}. In addition the PVA concentration can be reduced as low as 0.5 mg.mL^{-1}, in line with its expected IRI activity, then protein activity decreases. Considering the above PEG/PVA with 100 mg.mL^{-1} /1 mg.mL^{-1} respectively was used in the main manuscript. A serial of dilution of trehalose was also undertaken for comparison, figure S2.

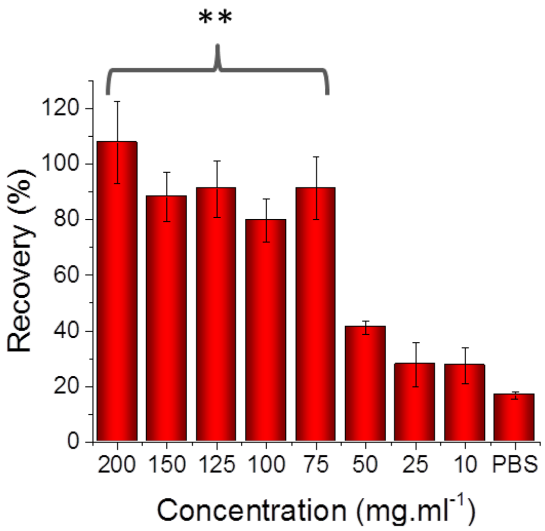

Figure S2. **Serial dilution of trehalose, frozen and stored for 3 days at -20 °C.** ** Represents p < 0.01. Error bars are calculated from a minimum of 6 repeats, recovery is expressed as a percentage of unfrozen β-Gal.

The ability of trehalose to protect β-Gal under freezing drops significantly below 75 mg.mL^{-1}. In comparison to PEG and PVA, which still provides significant recovery at 40 mg.mL^{-1} and 1 mg.mL^{-1} respectively. This is appealing as all additives can have potentially negative effects on biological materials when added in high concentration.

Assessment of Taq polymerase through qPCR

Curves of fluorescence against cycle number were plotted to determine a threshold value at which the fluorescence is highly significant against the background fluorescence as a way of measuring the efficiency of the Taq polymerase. The faster a sample reaches a certain threshold for example 500 fluorescence units the more active the Taq polymerase is, and hence the better the cryoprotection. In figure 4A, these threshold values are shown, so that easy comparison can be made.

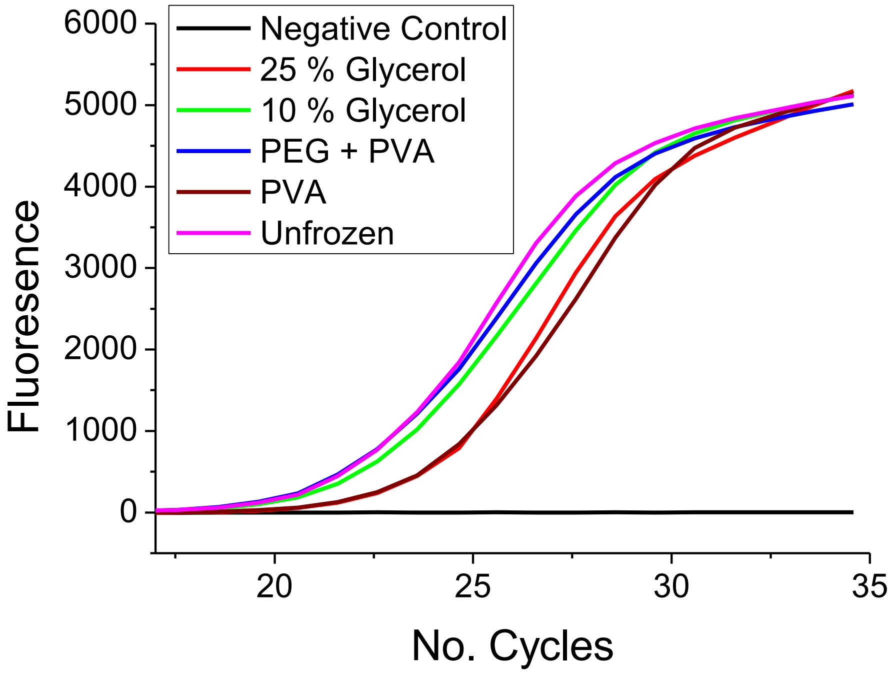

Figure S3. Quantitative PCR curves used to determine the threshold values (Figure 4A in main text).

Longer term storage at -20 °C of β-Gal.

Although long-term protein storage would normally be at -80 °C, cheaper -20 °C freezers (with more capacity) are more widely available and hence storage under these conditions was also assessed, Figure S4. Activity recovery was found to be significantly increased when the polymer mix was used when compared to PBS buffer for 1 week, but drops off after longer periods. Trehalose still maintains β-Gal activity over the full 4 weeks, suggesting that it would be of more use at this higher temperature, although it

has to be recognized that this is protein specific, as observed in the main body PEG and PVA provide better recovery for rabbit IgG.

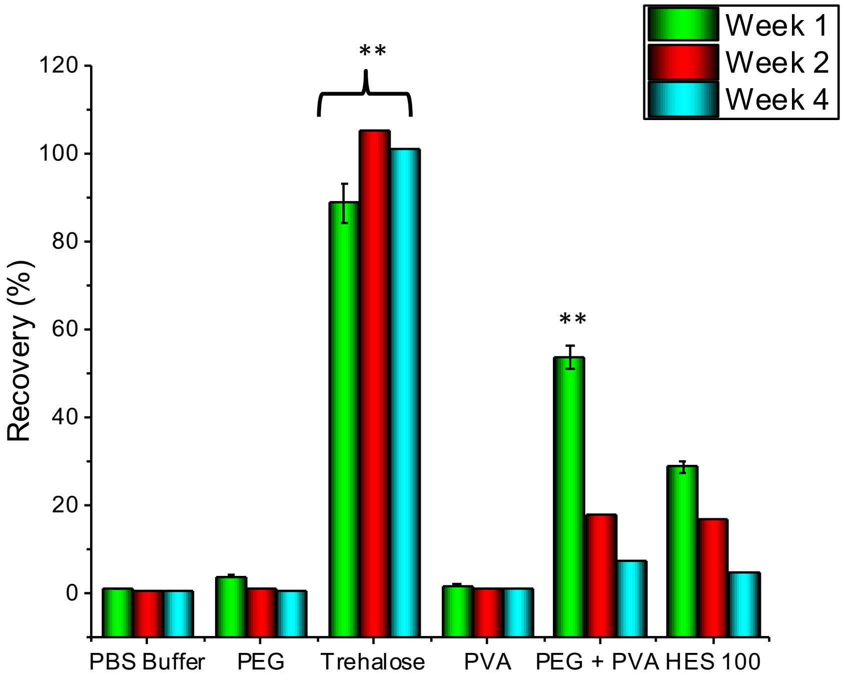

Figure S4. Longer term storage of β-Gal at -20 °C. Samples were thawed at room temperature (23 °C) after the indicated number of weeks stored at -20 °C. ** Represents $p < 0.01$. Error bars are calculated from a minimum of 6 repeats, recovery is expressed as a percentage of unfrozen β-Gal.

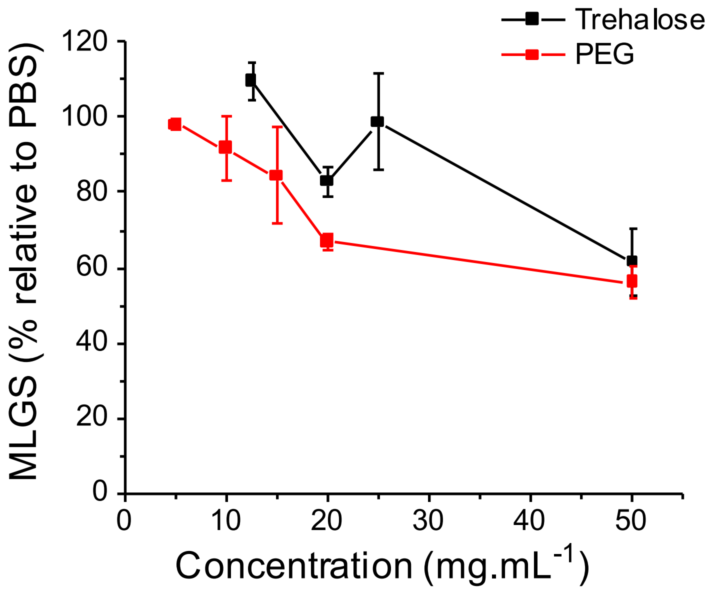

Figure S5. IRI activity of PEG and trehalose as a function of concentration. Data is reported as the average from n = 3, compared to a negative control of PBS alone. Error bars are +/- the standard deviation.

References

[1] a) D. E. Mitchell, M. Lilliman, S. G. Spain, M. I. Gibson, *Biomater. Sci.* 2014, *2*, 1787–1795; b) T. Congdon, R. Notman, M. I. Gibson, *Biomacromolecules* 2013, *14*, 1578-1586.

[2] A. S. G. Huggett, D. Nixon, *The Lancet* 1957, *270*, 368-370.
